# Expression of Stem Cell Markers in the Human Fetal Kidney

**DOI:** 10.1371/journal.pone.0006709

**Published:** 2009-08-21

**Authors:** Sally Metsuyanim, Orit Harari-Steinberg, Ella Buzhor, Dorit Omer, Naomi Pode-Shakked, Herzl Ben-Hur, Reuvit Halperin, David Schneider, Benjamin Dekel

**Affiliations:** 1 Department of Pediatrics and Pediatric Stem Cell Research Institute, Sheba Medical Center, Tel Hashomer, Sackler Faculty of Medicine, Tel Aviv University, Tel Aviv, Israel; 2 Department of Obstetrics and Gynecology, Assaf Harofe Medical Center, Zeriffin, Israel; Harvard Medical School, United States of America

## Abstract

In the human fetal kidney (HFK) self-renewing stem cells residing in the metanephric mesenchyme (MM)/blastema are induced to form all cell types of the nephron till 34^th^ week of gestation. Definition of useful markers is crucial for the identification of HFK stem cells. Because wilms' tumor, a pediatric renal cancer, initiates from retention of renal stem cells, we hypothesized that surface antigens previously up-regulated in microarrays of both HFK and blastema-enriched stem-like wilms' tumor xenografts (*NCAM, ACVRIIB, DLK1/PREF, GPR39, FZD7, FZD2, NTRK2*) are likely to be relevant markers. Comprehensive profiling of these putative and of additional stem cell markers (*CD34, CD133, c-Kit, CD90, CD105, CD24*) in mid-gestation HFK was performed using immunostaining and FACS in conjunction with EpCAM, an epithelial surface marker that is absent from the MM and increases along nephron differentiation and hence can be separated into negative, dim or bright fractions. No marker was specifically localized to the MM. Nevertheless, *FZD7* and *NTRK2* were preferentially localized to the MM and emerging tubules (<10% of HFK cells) and were mostly present within the *EpCAM^neg^* and *EpCAM^dim^* fractions, indicating putative stem/progenitor markers. In contrast, single markers such as *CD24* and *CD133* as well as double-positive *CD24^+^CD133^+^* cells comprise >50% of HFK cells and predominantly co-express *EpCAM^bright^*, indicating they are mostly markers of differentiation. Furthermore, localization of NCAM exclusively in the MM and in its nephron progenitor derivatives but also in stroma and the expression pattern of significantly elevated renal stem/progenitor genes *Six2, Wt1, Cited1, and Sall1* in NCAM^+^EpCAM^-^ and to a lesser extent in NCAM^+^EpCAM^+^ fractions confirmed regional identity of cells and assisted us in pinpointing the presence of subpopulations that are putative MM-derived progenitor cells (NCAM^+^EpCAM^+^FZD7^+^), MM stem cells (NCAM^+^EpCAM^-^FZD7^+^) or both (NCAM^+^FZD7^+^). These results and concepts provide a framework for developing cell selection strategies for human renal cell-based therapies.

## Introduction

Identification of multipotential progenitor populations in mammalian tissues is important both for therapeutic potential and an understanding of developmental processes and tissue homeostasis. Progenitor populations are ideal targets for gene therapy, cell transplantation, and tissue engineering of bioartificial organs(Weissman 2000; Xu et al. 2000). A demand for kidney progenitors is increasing because of a severe shortage of donor organs for orthotopic kidney transplantation. Because dialysis and kidney transplantation currently are the only successful therapies for patients suffering chronic renal failure, cell therapy with renal progenitors offers an alternative approach for therapies of kidney diseases(Dekel and Reisner 2006).

The early development of the mammalian metanephros, the direct precursor tissue of the adult kidney, is a complex process that involves highly regulated interactions between two derivatives of the intermediate mesoderm, the wolffian duct and the metanephric/nephrogenic mesenchyme. Reciprocal signaling between the metanephric/nephrogenic mesenchyme and a derivative of the nephric duct known as the ureteric bud results in branching of the ureteric bud (UB) and condensation of metanephric mesenchyme (MM) at its tips(Woolf 2001; Cho and Dressler 2003). The condensed mesenchyme is thought to form a precursor cell population, which both maintains itself at the tips of the UB (via proliferation and/or addition from the surrounding non-condensed mesenchyme) and gives off cells that differentiate into nephrons, the functional filtration unit of the kidney(Rosenblum 2008). Recent experiments have established that the progenitor cell in the MM fulfils the criteria of a true committed stem cell in that is capable of self-renewing and of differentiating towards different types of nephron epithelia(Self et al. 2006; Boyle et al. 2008; Kobayashi et al. 2008).

The human metanephros appears at the 5^th^ of gestation and renal stem/progenitor cells in the MM are induced to form nephrons until 34 weeks of gestation(Cho and Dressler 2003; Rosenblum 2008). For renal regeneration, both human precursor tissue(Dekel et al. 1997; Dekel et al. 2002; Dekel et al. 2003) or murine fetal kidney cell transplantation(Kim et al. 2007b; Kim et al. 2007a) can be utilized. Isolation of specific human renal progenitors from the MM requires the characterization of surface markers that would enable cell collection. Given the cellular heterogeneity in the developing human kidney(Rosenblum 2008), eliminating the unwanted mature cell populations from further cultivation steps, prior to transplantation, would increase the purity of the graft and allow for a better defined cell composition to be transferred.

While the transcriptional program specifying a renal progenitor cell has been thoroughly contemplated(Brodbeck and Englert 2004) corresponding cell surface markers have been hardly studied. Recently, we performed microarray studies of the human kidney, including fetal and adult kidneys (HFK and HAK, respectively) and their corresponding tumors, wilms' tumor (WT) and renal cell carcinoma (RCC) (Dekel et al. 2006b). Wilms' tumor is classified as a primitive, multilineage malignancy of embryonic renal precursors of the MM that are arrested in different stages of differentiation, thus forming in the tumor a cell population similar to condensed mesenchyme and also mature epithelial/tubular and stromal cells(Rivera and Haber 2005). While HFKs were heterogeneous, we used WT xenografts that by serial passage in mice were highly enriched for blastema at the expanse of differentiated elements(Dekel et al. 2006b; Metsuyanim et al. 2008). We were interested in genes that were up-regulated in both the stem-like WT xenografts and the HFK, as these were suggested to characterize the progenitor population arising from the MM (‘progenitor’ genes). Among these were the transcription factors specifying the kidney progenitor cells(Kreidberg et al. 1993; Nishinakamura 2003; Brodbeck and Englert 2004; Self et al. 2006) including *WT1, PAX2, LIM1, SIX1, EYA1, SALL1, and CITED1*. In addition, we detected cell surface markers, including *NCAM, ACVRIIB, FZD2, FZD7, GPR39, NTRK2* and *DLK1/PREF(Dekel et al. 2006b)*.

The aim of the present study was to determine the protein expression of these putative ‘cell surface progenitor markers’ in human fetal kidneys along with universal stem cell markers of various lineages so as to determine their relevance in characterization of renal stem/progenitor cells. Such characterization of putatively therapeutic cell suspensions according to surface markers is likely to be a prerequisite for future applications in a clinical setting.

## Materials and Methods

### Ethics Statement

This study was conducted according to the principles expressed in the Declaration of Helsinki. The study was approved by the Institutional Review Board of Sheba Medical Center, Wolfson hospital and Asaf Harofeh Medical Center hospitals. All patients provided written informed consent for the collection of samples and subsequent analysis.

### Establishment of a primary culture from human kidney

HFK were collected from elective abortions. Fetal gestational age ranged from 15 to 19 weeks. Normal HAK samples were retrieved from borders of RCC tumors from partial nephrectomy patients. This procedure was done following informed consent and has been approved by the local ethical committee. Collected tisuses were washed with cold HBSS (Invitrogen, Carlsbad, CA) and minced into ∼1 mm cubes using sterile surgical scalpels. The dissected tissue was then incubated for 2 hours in 37°C with Iscoves's Mod Dulbecco's Medium (IMDM) (Invitrogen) supplemented with 0.1% collagenase II (Invitrogen). The digested tissue was then gradually forced through a 100 µm, 70 µm and 50 µm cell strainer to achieve a single cell suspension, and after removal of the digesting medium resuspended in growth medium [IMDM containing 10% fetal bovine serum (Invitrogen), 100 ng/ml EGF, 100 ng/ml bFGF and 10 ng/ml SCF (R&D Systems, Inc, Minneapolis, USA)] and plated in flasks. Cells were incubated as described previously(Pode-Shakked et al. 2008 Nov in print) and used for FACS analysis of progenitor marker expression and sorting of subpopulations.. All assays were conducted with low-passage cultured cells (passage 1 or 2).

### Immunostaining of HFK

Immunostaining was performed as previously described ((da Silva Meirelles et al. 2006)) on three different kidneys: 12, 13, 18 weeks of human gestation. Briefly, 4 µm sections of HFKs were mounted on super frost/plus glass (Menzel, Glazer, Braunschweig, Germany) and processed by the labeled – (strept) avidin-biotin (LAB-SA) method using a histostain plus kit (Zymed). Heat-induced antigen retrieval was performed by controlled microwave treatment using an H2800 model processor (Energy Bean Sciences, INC) in 10 mM citrate buffer, PH 6.0 for 10 min at 970C. The sections were treated with 3% H2O2 for 10 min and stained for EZH2, CD56, CD90, DLK1, CD24, GPR39, CD133, SIX2, FZD7, FZD2, ACRIIB and NTRK1. Table 1 summarizes the antibodies used for immunostaining.

**Table 1:** Antibodies used for IHC staining.

Antibody	Marker Identified	Manufacturer	Catalog #
**Rabbit anti EZH2**	EZH2	Zymed, San Francisco, CA	SKU#36-6300
**Monoclonal anti-human CD56 (NCAM1)**	CD56	Ancell Corporation, Bayport, MN, USA	208-020
**Mouse anti-human CD90**	CD90, thy1	AbD serotec, Kidlington, Oxford, UK	MCA90
**Mouse anti- Preadipocyte factor-1**	DLK1, PREF1	Ray Biotec, Inc, Parkway Lane, Norcross GA	NR-08-0034
**Mouse monoclonal CD24**	CD24	abcam, Cambridge, UK.	ab31622
**Six2 monoclonal antibody**	SIX2	ABNOVA, Walnut, USA	H000010736-M01
**Rabbit polyclonal anti frizzled-7**	FZD7	NOVUS biologicals, Littleton, USA	NLS4900
**Rabbit polyclonal anti frizzled-2**	FZD2	NOVUS biologicals, Littleton, USA	NLS3488
**Monoclonal anti-human Activin RIIB antibody**	ACRIIB	R&D Systems, Inc, Minneapolis, USA	MAB3393
**Rabbit polyclonal to GPCR GPR39**	GPR39	abcam, Cambridge, UK.	ab39283
**Rabbit polyclonal to CD133**	CD133	abcam, Cambridge, UK.	ab16518
**Monoclonal anti-human TrkB antibody**	NTRK1	R&D Systems, Inc, Minneapolis, USA	MAB3971

Negative control incubations were performed by substituting non-immune serum for the primary antibody. Biotinylated second antibody was applied for 10 min followed by incubation with horseradish peroxidase –conjugated streptavidin (HRP-SA) for 10 min. Following each incubation, the slides were washed thoroughly with Optimax wash buffer (Biogenex, San Ramon, CA, USA). The immunoreaction was visualized by an HRP-based chromogen/substrate system, including DAB (brown) chromogen (liquid DAB substrate kit – Zymed). The sections were then counterstained with Mayer's hematoxylin, dehydrated and mounted for microscopic examination.

### Flow cytometry

FACS was performed on cultured cells originating from at least 3 independent samples ranging from 15 to 19 weeks of human gestation. Cells were detached from culture plated with non-enzymatic cell dissociation solution (Sigma-Aldrich Co., Ltd., St. Louis, MO) and a viable cell number was determined using Trypan blue staining (Invitrogen). Cells (1×10^5^ in each reaction) were suspended in 50 µl of FACS buffer [0.5% BSA and 0.02% sodium azid in PBS (Sigma-Aldrich and Invitrogen, respectively)] and blocked with FcR Blocking Reagent (MiltenyiBiotec, Auburn, USA) and human serum (1∶1) for 15 min at 4°C. Cells were then incubated for 45 min with a primary antibody for CD24, NCAM1, C-KIT, Thy-1, CD90, CD34, CD133, EpCAM, PSA-NCAM, ACVR2B, FZD7 or NTRK1 or a matching isotype control. All antibodies used for flow cytometry labeling are summarized in Table 2.

**Table 2:** Antibodies used in the flow cytometry assays.

Antibody	Marker identified	Isotype control	Manufacturer	Catalog #
**CD24-PE**	CD24	Mouse IgG1	eBioscience San Diego, USA	12-0247
**Biotin anti-human CD24**	CD24	Mouse IgG1	eBioscience	13-0247
**FITC anti-human CD34**	CD34	Mouse IgG2a	MiltenyiBiotec	130-081-001
**PE anti-human CD56 (N-CAM, NCAM1)**	NCAM1	Mouse IgG2a,κ	eBioscience	12-0569
**FITC mouse anti-human CD90**	Thy-1	Mouse IgG1,κ	BD Biosciences, San Jose, USA	555595
**CD133/1 (AC133)-APC**	CD133	Mouse IgG1	MiltenyiBiotec	130-090-826
**CD326 (EpCAM)- FITC**	EpCAM	Mouse IgG1	MiltenyiBiotec	130-080-301
**Monoclonal anti-human Activin RIIB antibody**	ACR2B	Mouse IgG1	R&D Systems, Inc.	MAB3393
**Biotinylated anti-human/mouse Frizzled-7 antibody**	FZD7	rat IgG2A	R&D Systems, Inc.	BAM1981
**FITC mouse anti-Human CD90**	CD90	Mouse IgG1,κ	BD Pharmingen	555595
**Affinity Purified antihuman CD117 (cKit)**	C-KIT	Mouse IgG1,κ	eBioscience	141179
**Monoclonal anti-human TrkB antibody**	NTRK1	Mouse IgG1	R&D Systems, Inc.	MAB3971
**Anti- PSA-NCAM-PE**	PSA-NCAM	Mouse IgM	MiltenyiBiotec	130-093-274

Cells were washed with FACS buffer, and incubated for 30 min at 4°C with a secondary Antibody if needed [Avidin-Fluorescein, APC Streptavidin or Alexa Fluor 647 goat anti mouse and Alexa Fluor 488 goat anti mouse. All secondary antibodies used are summarized in Table 3.

**Table 3:** Secondary Abs or S/A conjugated enzymes used in flow cytometry assays.

Reagent	Manufacture company	#
**Avidin-Fluorescein (Avidin-FITC)**	R&D Systems, Inc.	F0030
**APC Streptavidin**	BD Biosciences.	554067
**Alexa Fluor 647 goat anti mouse**	Invitrogen	A31625
**Alexa Fluor 488 goat anti mouse**	Invitrogen	A31620

Cell viability was tested using 7AAD viability staining solution (eBioscience). Cell labeling was detected using FACSCalibur (BD Pharmingen). Flow cytometry results were analyzed using FlowJo analysis software. Viable cells were defined by their FSC/SSC profiles and, in addition, their lack of 7AAD. Analysis of EpCAM subpopulations was performed by gating cell fractions according to EpCAM staining intensity (negative, dim or bright) versus FSC. The second marker was then examined in each subpopulation gate. When triple staining was performed, we initially gated EpCAM subpopulation and then examined co-staining of the other two markers in each subpopulation.

### Magnetic cell sorting

At least three independent kidney samples were used for sorting of NCAM/EpCAM as well as PSA-NCAM subpopulations. Sorted cells were of primary cultures established from the same HFK used in the FACS analysis of progenitor marker expression. Cells were detached with Trypsin/EDTA and resuspended in growth medium. Cells were transferred trough 30 µm Pre-Separation Filter (Miltenyi Biotec GmbH, Bergisch Gladbach, Germany) then washed and resuspended in pH 7.2 MACS buffer (0.5% BSA, 2 mM EDTA in PBSX1). Cells were magnetically labeled with NCAM1 (CD56) MultiSort MicroBeads kit (Miltenyi Biotec GmbH) according to the manufacturer's instructions and positive labeled cells (NCAM^+^) were enriched with LS Columns. CD56 MicroBeads were releases from the cells with MultiSort Release Reagent (Miltenyi Biotec GmbH) and CD56 positive cells were further separated with EpCAM positive and negative cells using CD326 MicroBeads (Miltenyi Biotec GmbH) on LS Columns according to the manufacturer's instructions. Enrichment of cells to CD56 and CD326 was validated using flow cytometry.

### Quantitative reverse transcription-PCR

NCAM^+^EpCAM^−^, NCAM^+^EpCAM^+^ and NCAM^−^ sub-populations of HFK were tested for the expression of: 1. Transcription factors specifying renal stem/progenitor cells in the MM (*SIX2, CITED1, SALL1, WT1, PAX2*) (5, 6), 2. The marker pair Vimentin/E-cadherin that are expressed in early stages of kidney development during mesenchymal (Vim+) to epithelial (E-cad+) conversion and differentiation (5, 6) 3. ‘Stemness’ genes (Wnt pathway, β-catenin; Polycomb group, *EZH2*, *BMI1*) 4. Pluripotency genes (*NANOG, OCT4*) and 5. Surface markers (ACR2B, FZD7, NTRK2, CD133 and CD24). In addition, sorted PSA-NCAM^+^ and PSA-NCAM^−^ HFK cells were analyzed for the expression of genes included in groups 1 and 2. Total RNA from cells was isolated using RNeasy Micro Kit (Qiagen GmbH, Hilden, Germany) according to the manufacturer's instructions. cDNA was synthesized using High Capacity cDNA Reverse Transcription kit (Applied Biosystems, California USA) on total RNA. Real-time PCR was performed using an ABI7900HT sequence detection system (Perkin-Elmer/Applied Biosystems) in the presence of TaqMan Gene Expression Master Mix (Applied Biosystems). PCR amplification was performed using gene specific TaqMan Gene Expression Assay-Pre- Made kits (Applied Biosystems). PCR results were analyzed using SDS RQ Manager 1.2 software. Statistical analysis was performed using a non-paired 2-tails T-test. Statistical significance was considered at P<0.05.

## Results

### Characterization of putative stem/progenitor markers in the HFK

#### SIX2

**Figure 1:**
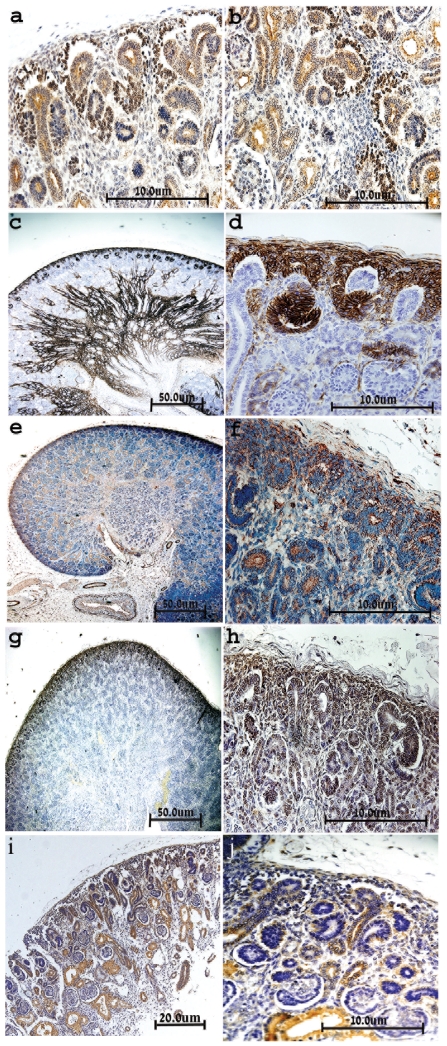
Immunostaining of putative stem cell markers. (a–j) Representative immunostaining of SIX2, NCAM1, FZD7, ACVR2B and NTRK2 in paraffin embedded sections of HFK (12, 13 and 18 weeks of human gestation); (a–b) localization of SIX2 to the MM, predominantly to the CM. (c–d) predominant staining of NCAM1 in the MM (including CM) and its derivatives (S- and comma- shaped bodies) and renal stroma, but not mature tubules or UBs. (e–f) FZD7 demonstrates preferential localization to the nephrogenic zone including MM and its derivatives, UBs, and newly forming tubules but not the stroma. (g–h) ACVRIIB immunostaining demonstrates predominant expression in the nephrogenic cortex; MM and its derivatives (S and comma shaped bodies), UBs, parietal epithelium of fetal glomeruli but not in the stroma. (i–j) NTRK2 is detected in the MM (including condensates) and its derivatives, UBs and some differentiated tubules. Figures c, e and g are shown in low magnification (original×4), Figures a, b, d, f and h–j are shown in higher magnifications (original×40; I, original×20).

Of the multiple regulatory genes specifying renal progenitors, SIX2 is a transcription factor that has been shown in mice to specify self-renewing epithelial renal stem cells that have the ability to give rise to all cell types in the nephron(Rosenblum 2008). Immunostaining of mid-gestation HFK revealed localization of such SIX2-expressing cells to the MM, specifically to the cap mesenchyme (CM), where renal stem cells are suggested to reside(Boyle et al. 2008; Rosenblum 2008) (Fig. 1a–b). While unsuitable for human cell sorting, SIX2 staining highlights the location of the desired putative MM stem cells.

#### EpCAM (CD326)

**Figure 2:**
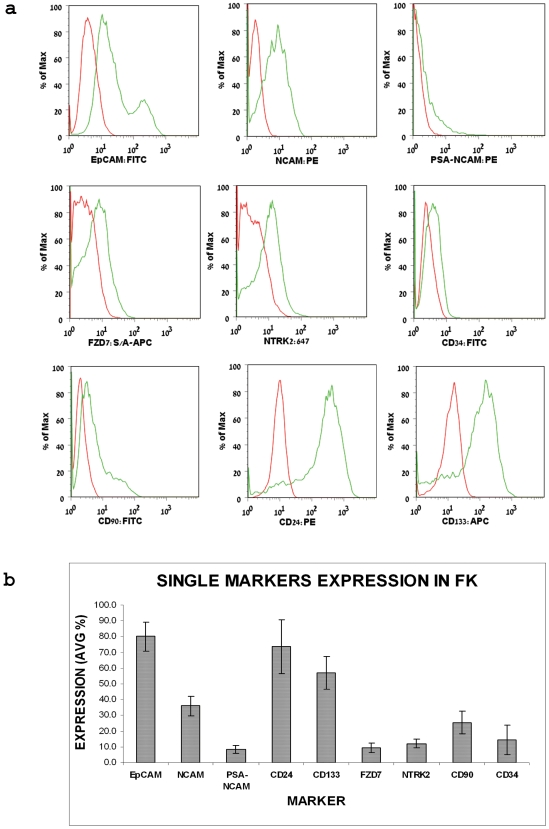
FACS analysis of single putative stem cell surface markers in HFK cells. (a) Representative flow-cytometry histograms of surface marker molecules (green) EpCAM, NCAM1, FZD7, NTRK2, CD90, CD34, CD24, CD133, and their respective isotype controls (red) in HFK (19 weeks of gestation). (b) Summarizing bar graph of single marker staining in HFK (15–19 weeks of gestation). Each marker was tested on at least 3 independent samples. Data were calculated as average % of expressing cell±SD. Each marker was tested in 10 HFK.

**Figure 3:**
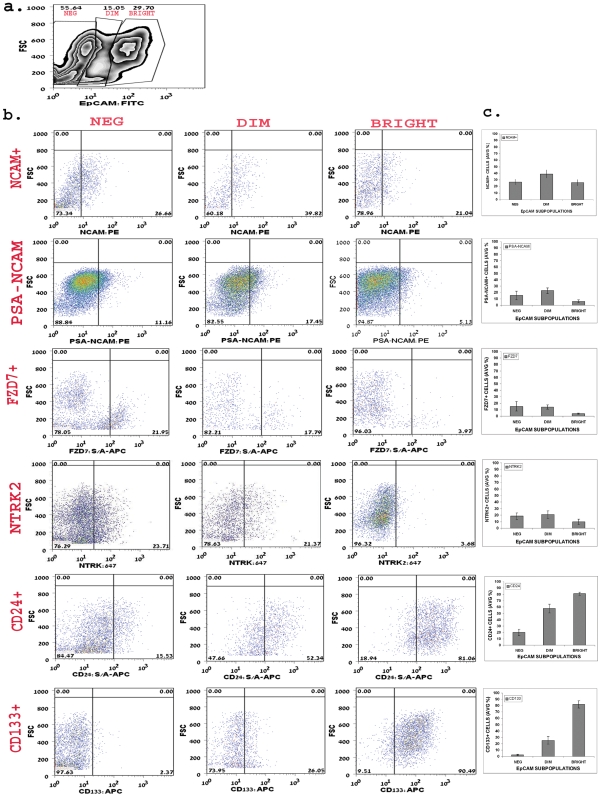
FACS analysis of putative stem cell markers in EpCAM subpopulations. (a) Representative zebra graph of EpCAM staining and the subpopulation gating. EpCAM subpopulations were gated according to EpCAM staining intensity (negative, dim or bright) versus FSC. (b) Representative dot plot graphs of NCAM1, PSA-NCAM, FZD7, NTRK2, CD24 and CD133 expression levels in EpCAM subpopulations of HFK. Quadrates were placed according to isotype control confiding the negative staining to the lower left quadrant. Percentage of cells in each subgroup appears on the lower right quadrant. (c) Summarizing bar graphs of NCAM1, PSA- NCAM, NTRK2, FZD7, CD24 and CD133 expression levels in EpCAM subpopulations. Data are average % of cells in each subgroup±SD. Analysis of each marker was performed at least three times.

The Epithelial Cell Adhesion Molecule (EpCAM) is expressed virtually on all normal epithelia in vertebrates(Trzpis et al. 2008) and can therefore serve as a marker for epithelial differentiation. Accordingly, Trzpis et al(Trzpis et al. 2007) have recently shown that in mid-gestation HFK (by 10 weeks of gestation), hEpCAM was expressed by the ureteric bud (UB) and comma-shaped (C) and S-shaped (S) bodies, whereas the MM did not express hEpCAM. Moreover, they found differential hEpCAM staining levels during nephrogenesis, where the weakest staining for hEpCAM was observed in the C- and S-shaped bodies, which are progenitor nephron derivatives of the MM and higher levels in the UB and developing tubules of the nephron, indicating a correlation between hEpCAM levels and the degree of epithelial differentiation. We examined cell populations of low-passage HFK cells by flow cytometry and revealed that 80.0±11.2% of the cells express EpCAM (Fig. 2a–b). This result correlated with its wide spread distribution in epithelial cells of the developing kidney. Moreover we detected two subpopulations within the EpCAM^+^ population, EpCAM^dim^ and EpCAM^bright^, suggestive of epithelial progenitor and more differentiated tubular cells, respectively (Fig. 3a). A clearer separation between EpCAM^dim^ and EpCAM^bright^ cell populations was noted in older HFK.

#### NCAM1 (CD56)

**Figure 4:**
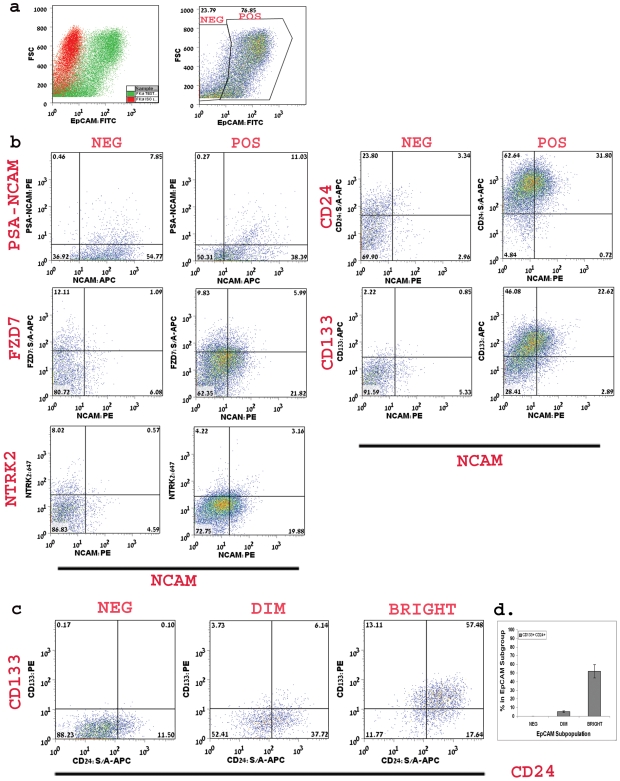
FACS analysis of putative stem cell markers in NCAM/EpCAM subpopulations. (a) Representative dot plot graphs of EpCAM staining. Cells were gated in two groups: EpCAM negative (neg) and EpCAM positive (pos) versus FSC. (b) Representative dot plot graphs of PSA- NCAM, FZD7, NTRK2, CD24 and CD133 co-staining with NCAM in EpCAM positive or negative populations of mid-gestation HFK. Quadrates were placed according to the isotype control confiding the negative staining to the lower left quadrant. Percentage of cells for each quadrant appears in the quadrant. (c) Representative dot plot graphs of CD24 and CD133 co-staining in EpCAM subpopulations of HFK. Quadrates were placed according to the isotype control confiding the negative staining to the lower left quadrant. Percentage of cells for each marker combination appears in the quadrant. (d) Summarizing bar graphs of CD24 and CD133 co-staining in EpCAM Subpopulations. Data are average % of cells in each subgroup±SD. Analysis of each marker was performed at least three times.

Transcript levels were up-regulated in both HFK and stem-like WT xenografts (>three-fold increment) (Dekel et al. 2006b). Immunostaining of sections of HFK (14–20 week) demonstrated predominant staining in the nephrogenic zone and renal stroma, while mature tubules were devoid of staining. In the nephrogenic zone, we observed strong expression in the CM, similar to *SIX2* and also in early S and C shaped nephron figures (i.e., MM and its derivatives) and newly forming tubules but not in UBs (Fig. 1c–d). This staining pattern of NCAM has been observed in the developing mouse kidney(Klein et al. 1988; Bard et al. 2001). Examination of populations HFK cells by single staining flow cytometry revealed that 29.1±8.2% of the cells express NCAM (Fig. 2a–b), representing nephrogenic zone and stroma-derived NCAM expressing cells. We further detected two sub-populations of NCAM^+^ cells, NCAM^+^EpCAM^−^ (13.5±4.9% of total cells) and NCAM^+^EpCAM^+^ (14.5±3.7% of total cells). Because EpCAM is not expressed in the stroma or in the MM, the NCAM^+^EpCAM^−^ subpopulation is indicative of cells originating from both of these areas, while NCAM^+^EpCAM^+^ cells are a heterogeneous pool of progenitor cells from the nephrogenic zone, including newly developed tubules. This sub-population could be further separated into NCAM^+^EpCAM^dim^ and NCAM^+^EpCAM^bright^ cell fractions (Fig. 3). In the EpCAM^dim^ population a significantly larger fraction consists of NCAM expressing cells compared to that found in the EpCAM^bright^ cell fraction (P<0.0001) (Fig. 3b), further indicating NCAM as an epithelial progenitor marker. Taking into account that in the nephrogenic zone low levels of EpCAM were previously noted in the immediate MM-derived structures (S- and C-shaped) and higher levels in emerging tubules, the NCAM^+^EpCAM^dim^ cells possibly represent the former. In addition, we have analyzed the long chain form of polysialic acid (PSA) characteristic of the low adhesive embryonic form of NCAM (PSA-NCAM), representing a post-translational modification and therefore relevant at the protein level. This surface marker closely resembles NCAM's staining pattern (various developmental stages including condensed MM, renal vesicles, the distal portion of S-shaped bodies, and primitive tubules) but is not detected in the renal stroma(Roth et al. 1988). Accordingly, we found PSA-NCAM to be expressed in 8.6±3.2% of HFK cells (2a–b) and to peak in the EpCAM^dim^ cell fraction (P<0.015 compared to the EpCAM^bright^ cell fraction) (Fig. 3b). Furthermore, PSA^+^EpCAM^−^ and PSA^+^EpCAM^+^ cell fractions are more limited in expression by comparison to NCAM/EpCAM (2.3±1.3% and 4.2±0.9% of total cells, respectively). Interestingly, when applying triple staining for PSA-NCAM, NCAM and EpCAM we found the putative MM cell fraction, NCAM^+^PSA-NCAM^+^EpCAM^−^, to be expressed in 2.5±2.2% of total cells, while NCAM^+^PSA-NCAM^+^EpCAM^+^ from later developmental stages in 4.3±0.3% of total cells (Fig. 4), indicating that PSA-NCAM and NCAM localize in similar progenitor areas. We could not detect NCAM^−^PSA-NCAM^+^EpCAM^−^ cells.

#### Frizzled 2,7 (FZD2, FZD7)

**Figure 5:**
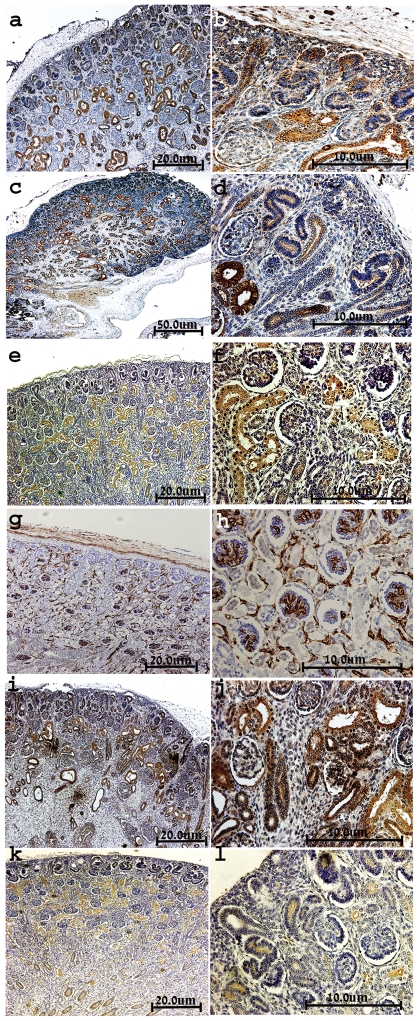
Immunostaining of putative stem cells markers. (a–l) Representative immunostaining of FZD2, GPR39, DLK1, CD34, CD90 and CD24 in paraffin embedded sections of HFK (12, 13 or 18 weeks of gestation); (a–b) FZD2 immunostaining demonstrates widespread staining of renal tubules. (c–d) GPR39 immunostaining demonstrates ubiquitous expression in differentiated renal tubular and to a lesser extent in componenets of the nephrogenic cortex. (e–f) Dlk1 immunostaining demonstrates ubiquitous expression in differentiated renal tubular but not in MM and its derivatives renal, UBs or stroma. (g–h) CD34 immunostaining demonstrates exclusive localization to endothelial cells (glomerular and peri-tubular) in all parts of the HFK, including in the nephrogenic cortex. (i–j) CD90 immunostaining demonstrates predominant staining in renal tubular cells but not in MM and its derivatives, UBs or stroma. (k–l) CD24 immunostaining demonstrates widespread expression in mature tubules. Figure (c) is shown in low magnification (original×4), Figures a, e, g, i, k and b, d, f, h, j, l are shown in higher magnifications (original×20 and×40, respectively).

Both transcript levels of *FZD2* and *FZD7* (Wnt receptors) were up-regulated in both HFK and stem-like WT xenografts(Dekel et al. 2006b). Recently, activation of the Wnt/β-catenin pathway has been shown to maintain the progenitor pool in the MM(Schmidt-Ott and Barasch 2008). Thus, FZD proteins represent surface marker molecules that may have a functional role in maintaining progenitor cells. Immunostaining HFK revealed that while FZD2 demonstrated widespread expression (Fig. 5a–b), staining all of the tubular cells, FZD7 was detected predominantly in the nephrogenic zone, staining all cell types in that area [MM (both loose and condensed mesenchyme), UBs, early nephron figures, newly forming tubules] but not at all in renal stroma (Fig. 1e–f). Correlating with its reserved localization, FZD7 was detected in only 9.5±3.7% of the HFK cells (Fig. 2a–b). Examination of the FZD7 expressing cells in relation with EpCAM sub-populations, showed that largest fractions of FZD7^+^ cells exists within the EpCAM^neg^ and EpCAM^dim^ fraction and to a much lesser extent in the EpCAM^bright^ cell fraction (P<0.02) (Fig. 3b). Thus, while EpCAM^+^FZD7^−^ cells represent the largest fraction (53.8±13.4% of total cells), we observed FZD7^+^EpCAM^+^ cells (3.9±1.2% of total cells) which likely represent MM- and UB- derived progenitors and FZD7^+^EpCAM^−^ cells (2.5±0.6% of total cells), which may originate solely from the MM. Furthermore, using triple FACS staining of HFK cells that also includes NCAM (Fig. 4b) we were able to demonstrate cell populations of the FZD7^+^EpCAM^+^NCAM^+^ progenitor phenotype (MM-derived, 2.5±1.0% of total cells, 4.7±1.0% FZD7^+^NCAM^+^ cells within the EpCAM^+^ population) as well as FZD7^+^NCAM^+^EpCAM^−^ (0.6±0.5% of total cells, 2.2±0.7% FZD7^+^NCAM^+^ cells within the EpCAM^−^ population) and surprisingly also FZD7^+^EpCAM^−^NCAM^−^ phenotypes (2.0±0.8% of total cells, 7.8±4.18% FZD7^+^NCAM^−^ cells within the EpCAM^−^ population), which are both likely to represent putative MM-originating stem cells.

#### Activin receptor IIB (ACVRIIB)

ACVRIIB qualified as a microarray predicted marker. Interestingly, mice lacking ACVRIIB show abnormalities in kidney development and in anterior/posterior patterning of the axial skeleton show abnormalities(Oh and Li 1997; Esquela and Lee 2003), further emphasizing functional importance in the renal progenitor population. Similar to NCAM and FZD7, in the sections of HFK, ACVRIIB was preferentially localized to the nephrogenic zone, showing strong expression in all structure types (blastema, UBs, C- and S-shaped bodies and also developing tubules). ACVRIIB was also detected in parietal epithelium of fetal glomeruli but not on stromal cells (Fig. 1g–h). A similar staining pattern was observed by in-situ hybridization of E14.5 mouse kidneys (robust expression of *ActRIIB* mRNA in the condensed MM, differentiating nephrons and UB branches). While according to its localization ACVRIIB has potential as a renal progenitor marker, FACS analysis of HFK cells showed extremely varying expression levels and precluded its further investigation.

#### NTRK2

NTRK2 qualified as a microarray predicted marker as similar to FZD7 it was up-regulated in microarrays of WT-stem like tumors and HFK. Previous analysis of the developing mouse kidney showed NTRK2 to localize to the MM while in WT NTRK2 has been suggested as a bad prognostic marker(Durbeej et al. 1993). Immunostaining of the HFK showed NTRK2 to localize to cells within the MM but also to early differentiation stages in the nephrogenic zone and some differentiated tubules but not stroma (Fig. 1i–j). FACS analysis revealed NTRK2 to stain 12.1±3.4% of the HFK (Fig. 2a–b). Analysis of NTRK2 according to EpCAM subpopulations revealed a tendency towards higher expression levels in both the negative and dim fraction compared to the bright one (Fig. 3b). To further strengthen the presence of progenitor phenotypes we found by triple staining of NTRK2 along with NCAM and EpCAM, EpCAM^+^NCAM^+^NTRK2^+^ cells (3.1±2.5% of total, 6.8±3.3% NCAM^+^NTRK2^+^ cells within the EpCAM^+^ population) as well as putative MM stem cell populations, EpCAM^−^NCAM^+^NTRK2^+^ cells (0.61±0.3% of total, 3.3±2.5% NCAM^+^NTRK2^+^ cells within the EpCAM^−^ population) and EpCAM^−^NCAM^−^NTRK2^+^ cells (2.7±2.4% of total, 7.5±2.7% NCAM^−^NTRK2^+^ cells within the EpCAM^−^ population) (Fig. 4b).

#### GPR39, DLK1

These markers, up-regulated in microarrays of both HFK and stem-like WT xenografts, were found to be ubiquitously expressed in differentiated renal tubular epithelial cells in sections of HFK while only faintly positive or negative in progenitor structures of the nephrogenic zone and were therefore eliminated from FACS analysis (Fig. 5c–d and e–f, respectively).

#### CD34

CD34 is a well known marker of hematopoietic stem cells (HSC) (Dekel et al. 2006a). FACS analysis demonstrated CD34 to be expressed in 14.4±12.9% of HFK cells (Fig. 2a–b). Immunostaining for the CD34 protein specifically demonstrated widespread endothelial localization (glomerular and peri-tubular) in all parts of the HFK (Fig. 5g–h), including in the nephrogenic zone whereas CM and other epithelial progenitor structures are devoid of CD34 expression. CD34 is therefore not an epithelial stem cell marker in the HFK but rather a marker for vascular differentiation. c-Kit, an additional hematopoietic stem cell marker, was not detected in the HFK cells.

#### CD90

Antigenic phenotypes of adult MSC consistently include CD90 and CD105(Dominici et al. 2006). In addition, CD90 was shown to be broadly expressed on heterogeneous rat FK cells transplanted to injured kidneys(Kim et al. 2007b). Immunolocalization of CD90 in the HFK revealed predominant expression in renal tubular cells but not in the nephrogenic zone (Fig. 5i–j) and 25.3±8.5% of HFK cells expressed CD90.

#### CD24

CD24 was not differentially expressed in the developing human kidneys or in WT stem-like xenografts. Nevertheless, the previous demonstration of CD24 as characteristic of the molecular phenotype of renal progenitor cells in the developing mouse kidneys(Challen et al. 2004), as well as the utilization of CD24 (along with CD133) to specify human renal progenitor cells(Lazzeri et al. 2007) from developing human kidneys, have led us to examine its expression. Immunostaining of HFK showed widespread expression and localized CD24 to mature tubules (renal stroma was devoid of CD24) (Fig. 5k–l). Accordingly, FACS analysis demonstrated that approximately 73.6±20.6% of HFK cells express CD24 (Fig. 2a–b). When analyzed in regard with EpCAM sub-populations, the abundance of CD24 expressing cells increases along epithelial differentiation (in contrast with for instance FZD7) so that approximately 80% of the EpCAM^bright^ cells are CD24^+^ cells (P<0.0001 compared to CD24^+^ cells found in the dim and negative fractions) (Fig. 3b), indicating that CD24 is predominantly a marker of differentiation in the HFK. Moreover, triple staining with NCAM revealed that CD24 is expressed in low levels only in putative MM fractions; CD24^+^NCAM^+^EpCAM^−^ and CD24^+^NCAM^−^EpCAM^−^ cell fractions (2.0±1.2%, 3.7±2.8% of total cells, respectively) in contrast to a CD24^+^NCAM^−^EpCAM^+^ differentiated phenotype (34.1±14.6% of total cells) (Fig. 4b). Thus, sorting cells from the HFK according to CD24 would result in a heterogeneous population comprised predominantly of differentiated cells and to a much lesser extent of stem/progenitor cells.

#### CD133

Although the biological function of CD133 remains unknown, CD133 is recognized as a stem cell marker for normal and cancerous tissues(Shmelkov et al. 2008). Indeed, CD133 alone or in a combination with other markers is currently used for the isolation of stem cells from numerous tissues, such as bone marrow, brain, prostate, liver, pancreas(Uchida et al. 2000; Salven et al. 2003; Sugiyama et al. 2007; Shmelkov et al. 2008), and both developing and adult kidney (along with CD24)(Sagrinati et al. 2006; Lazzeri et al. 2007). Among adult organs, the kidney has been reported to have large numbers of CD133^+^ cells^35^, (Weigmann et al. 1997). As previously shown for the fetal pancreas, we could not detect CD133 positivity in HFK tissue. However, FACS analysis of HFK cells demonstrated that 56.9±15.8% of the cells express CD133 (Fig. 2a–b). Furthermore, the EpCAM^bright^ fraction contained the largest population of CD133 expressing cells with significantly smaller populations in EpCAM^dim^ and EpCAM^neg^ cells (P<0.0001) (Fig. 3b). In addition, similar to CD24, triple FACS staining demonstrated a large population of CD133^+^EpCAM^+^NCAM^−^ cells (29.5±10.6% of total cells) and a relatively small ones of the CD133^+^NCAM^+^EpCAM^+^ (14.4±4.5% of total cells) and CD133^+^NCAM^+^EpCAM^−^ putative progenitor and stem phenotypes (1.1±1.2% of total cells) (Fig. 4b). Because CD24^+^CD133^+^ cells have been recently suggested a renal ‘stem cell’ fraction(Lazzeri et al. 2007), we analyzed expression of CD133 in conjunction with CD24. Double staining showed that the CD24^+^CD133^+^ fraction comprises 55.5±6.4% of the HFK cells, while triple staining with EpCAM showed that within the EpCAM^bright^ fraction approximately 60% of the cells are CD24^+^CD133^+^ and to a much lesser extent in the EpCAM^dim^ and EpCAM^neg^ cell fractions (P<0.0001) (Fig. 4c). Thus, similar to cells expressing the CD24 marker, most of the CD133^+^ cells in the HFK and also CD133^+^CD24^+^ cells are of a differentiated tubular phenotype and are not likely to be exclusive to the stem/progenitor pool.

#### HFK cell sub-populations retain molecular aspects of regional identity

**Figure 6:**
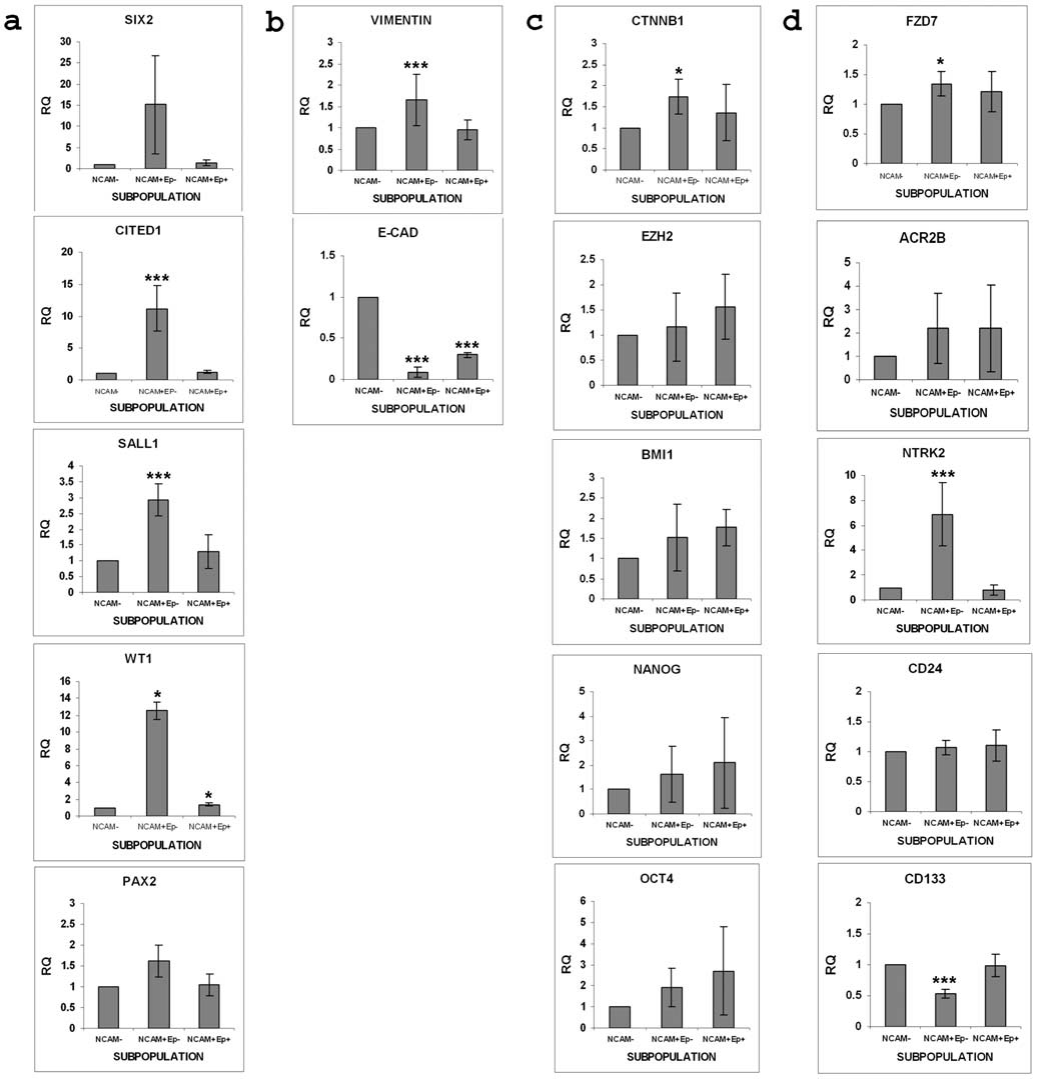
Gene expression analysis in sorted NCAM/EpCAM subpopulations. Quantitative reverse transcription-polymerase chain reaction (qRT-PCR) analysis of (a) renal stem/progenitor genes (*Six2, Cited1, Sall1, Wt1* and *Pax2*), (b) vimentin and E-cadherin (c) ‘stemness’ genes (β-catenin/CTNNB1, *EZH2*, *BMI1*, *Nanog* and *Oct4*) and (d) surface marker (*FZD7, ACR2B, NTRK2, CD24* and *CD133*) gene expression in NCAM/EpCAM magnetically separated cells from HFK (15–19 weeks of gestation). Normalization was performed against control HPRT expression and RQ calculated relative to the NCAM- fraction. Data were calculated as average±SD of at least 3 independent samples. ***P<0.001, *P<0.05 versus NCAM-. *Sall1* expression in NCAM^+^ EpCAM^+^ cells was near significance (p<0.059).

**Figure 7:**
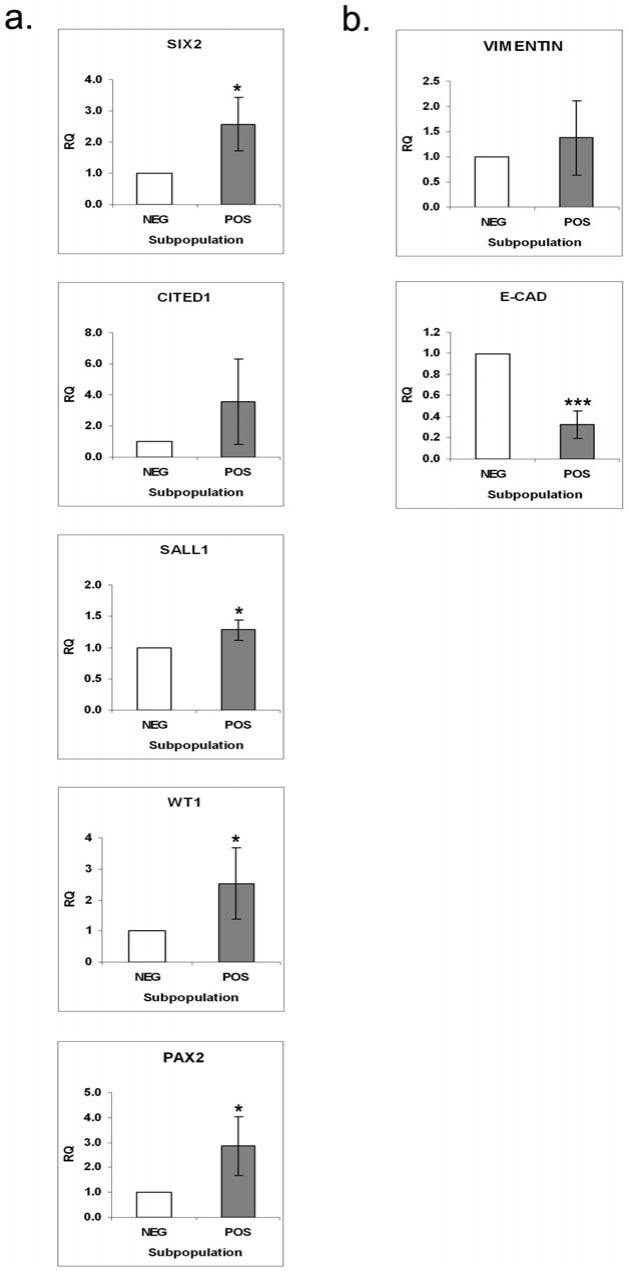
Gene expression analysis in sorted PSA-NCAM subpopulations. Quantitative reverse transcription-polymerase chain reaction (qRT-PCR) analysis of (a) renal stem/progenitor genes (*Six2, Cited1, Sall1, Wt1* and *Pax2*), (b) vimentin and E-cadherin genes expression in PSA-NCAM magnetically separated cells from HFK (15–19 weeks of gestation). Normalization was performed against control HPRT expression and RQ calculated relative to the PSA-NCAM^−^ fraction. Data were calculated as average±SD of at least 3 independent samples. ***P<0.001, *P<0.05 versus PSA-NCAM^−^. *Sall1* expression in NCAM^+^ EpCAM^+^ cells was near significance (p<0.059).

Because immunostaining of HFK demonstrated that markers are regionally specified we wanted to verify that regional differences are maintained in HFK cells in addition to FACS separation according to EpCAM intensity levels. As a proof-of-principle we chose to analyze sorted NCAM^+^EpCAM^−^, NCAM^+^EpCAM^+^ (containing putative MM stem- and MM-derived progenitor cells, respectively) in comparison with NCAM^−^ HFK cell populations as NCAM and EpCAM are important surface markers for our characterization system. Although a heterogeneous cell population (see before), NCAM^+^EpCAM^−^ cells highly overexpressed (>five fold) most MM stem/progenitor genes in five separate HFK (Fig. 6a), levels of which were already reduced in the NCAM^+^EpCAM^+^ cell fraction (presumably more differentiated), but still higher (*Wt1*, *Sall1*) in comparison with the NCAM^−^ cell fraction (Fig. 6a), indicating a hierarchy for enrichment for the renal ‘progenitor’ genes. Considerably lower E-cad levels were observed for the NCAM^+^EpCAM^−^ and NCAM^+^EpCAM^+^ cell fractions, while NCAM^+^EpCAM^−^ also significantly overexpressed vimentin (Fig. 6b). In addition, while there was a tendency for elevation of the ‘stemness genes’ in the NCAM^+^ fractions, only β-catenin achieved significance in NCAM^+^EpCAM^−^ cells (Fig. 6c), most likely due to large variations across human samples. Finally, analysis of surface marker expression in the sorted sub-populations showed elevated FZD7, ACVRIIB and NTRK2 in the NCAM^+^ fractions (both *FZD7* and *NTRK2* genes significantly overexpressed in the NCAM^+^EpCAM^−^ fractions) as opposed to CD24 and especially CD133 (Fig. 6d). Similar results were found when analyzing expression in sorted PSA-NCAM^+^ cells by comparison to the negative fraction. PSA-NCAM (see before) showed significant enrichment for *Six2*, *Sall1*, *Wt1* and *Pax2* (Fig. 7a) as well as reduced levels of E-cadherin (Fig. 7b), all indicative of a stem/progenitor origin. Thus, HFK cells retain aspects of regional identity as determined by marker immunostaining.

#### Marker expression in the human adult kidney (HAK)

**Figure 8:**
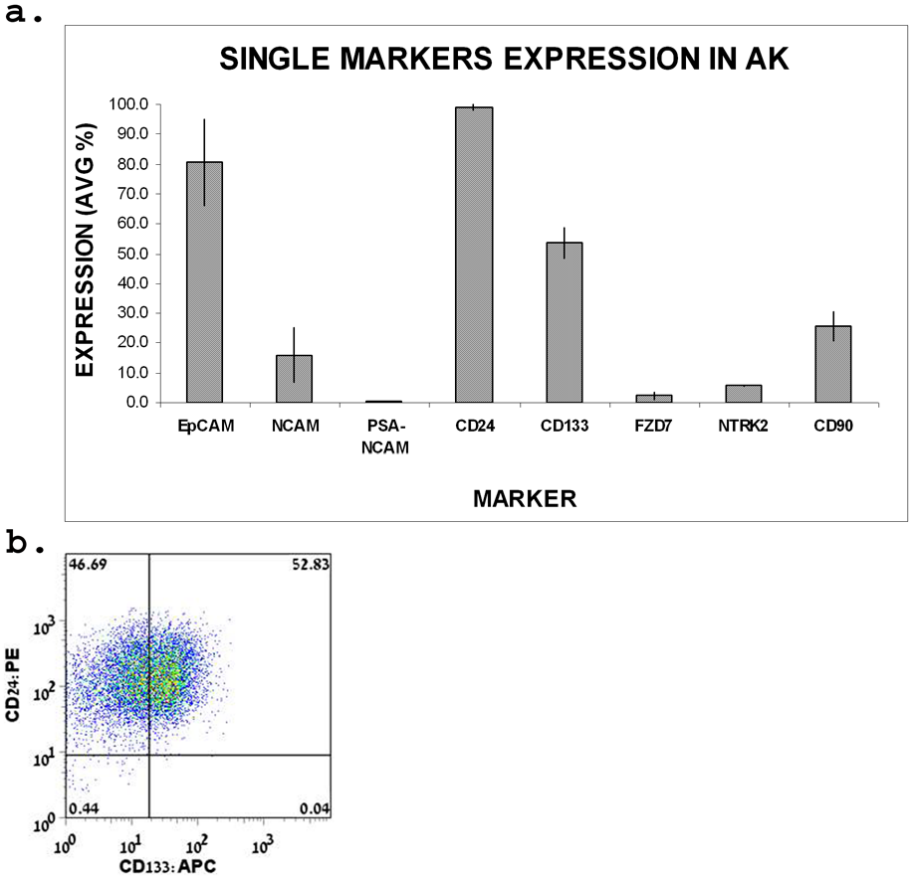
FACS analysis of single putative stem cell markers in HAK cells. (a) Summarizing bar graph of single marker staining in HAK cells. Data were calculated as average % of expressing cell±SD. Each marker was tested in 3 HAK. (b) Representative dot plot graphs of CD24 and CD133 co-staining demonstrate a large fraction of CD24^+^CD133^+^ cells in HAK. Quadrates were placed according to the isotype control confiding the negative staining to the lower left quadrant. Percentage of cells for each marker combination appears in the quadrant.

Renal cell progenitor markers are expected to decrease once maturation occurs. We therefore analyzed cell surface marker expression in the HAK. Indeed, flow cytometry analysis of HAK cells for single marker expression revealed reduced PSA-NCAM, FZD7, NTRK2 and NCAM levels compared to HFK, indicative of a progenitor origin (Fig. 8a). In contrast, similar and even increased expression levels in the HAK were observed for CD105, CD90, CD133 and CD24 (Fig. 8a). Moreover, CD24^+^CD133^+^ cells represent a large cell fraction in the HAK, comprising 64.26±10.15% of the total cells (Fig. 8b), precluding these markers from exclusively specifying a progenitor pool.

## Discussion

In the present study, we have analyzed for the expression of putative stem cell markers in the in the human fetal kidney. Using comprehensive immunocytochemical and flow cytometric analysis of HFK cells, we characterized the expression profile of a variety of surface antigens some of which are considered markers of organ-specific stem cells and the others have been recently suggested by us to appear on malignant renal stem/progenitor cells of wilms' tumors and in the developing human kidney(Dekel et al. 2006b). Given the similarities in molecular marker expression in progenitors from wilms' tumors and the developing human kidney, it appears likely that these cell populations are derivatives of the same lineage. Our data suggest that none of these putative stem cell markers are restricted to kidney-specific epithelial stem/progenitor cells, but on the contrary, stem cell markers are always also expressed on differentiated elements. The necessity for marker combination is shown not only by lack of specific staining of the nephrogenic mesenchyme but also by high percentage of expression of single markers in HFK cells, over 50% of cells for markers such as CD24 and CD133, as well as the relative high marker abundance within the EpCAM^bright^ fraction and persistence in high levels in the HAK. Because CD24 and CD133 mostly qualify as markers for identification of differentiated tubular cells, their combination will not enrich for a progenitor phenotype. Thus, areas previously reported to contain renal stem cells in the adult kidney [35] might not necessarily originate from stem cells but rather contain differentiated cells with clonogenic proliferating capacities as recently shown for pigmented ciliary epithelial cells (Cicero et al. 2009).

**Figure 9:**
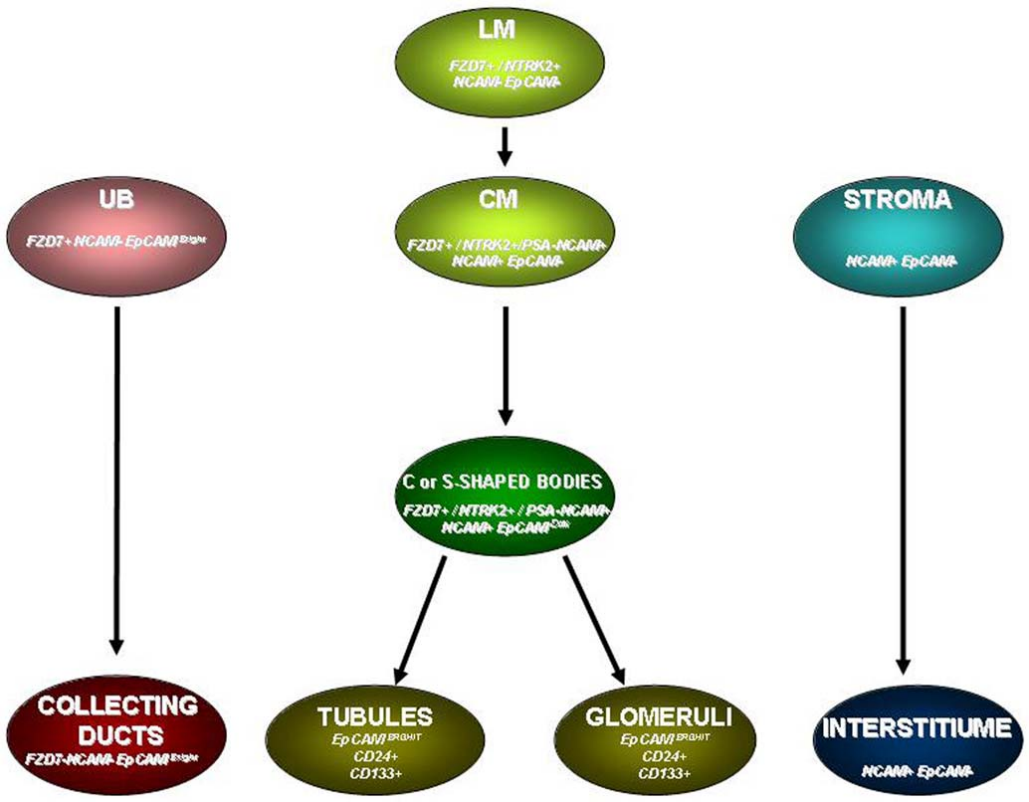
Changes in surface marker expression along renal progenitor differentiation. A hypothetical model of changes in surface marker expression along differentiation of the nephric-lineage relevant for isolation of cell subpopulations. Abbreviations: MM, metanephric mesenchyme; CM, condensed mesenchyme; LM, loose mesenchyme; UB, ureteric bud.

More relevant for the enrichment of stem/progenitor cells is the utilization in combination of at least one of the markers that were found to localize predominantly to the nephrogenic zone and to a much lesser extent to differentiated epithelia (NCAM, PSA-NCAM, FZD7, and NTRK2). Moreover, these markers are less abundant in HFK cells and decline in the adult kidney. The maintenance of regional identity of HFK cells was exemplified by these markers' abundance in EpCAM^neg^ and EpCAM^dim^ fractions as well as by analyzing sorted NCAM expressing cell subpopulations (including its embryonic form, PSA-NCAM) and showing mostly highly significant differences in genes that are well-established early markers of kidney stem/progenitor cells (*Six2, Wt1, Pax2, Cited1, and Sall1*). Interestingly, we have recently identified NCAM as a candidate marker for the renal malignant progenitor population of wilms' tumor(Pode-Shakked et al. 2008 Nov in print). Because NCAM is not at all expressed on UBs or differentiated epithelia it can be extremely useful for positive selection of MM-derived progenitor nephron populations (NCAM^+^X^+^) if the second marker is clearly not detected on MM and stromal cells. This definition is most suitable for the NCAM^+^EpCAM^+^ fraction we detected among the HFK cells and shown to enrich, at least in part, for the renal ‘progenitor’ genes. Moreover, because EpCAM is differentially expressed in the nephrogenic zone(Trzpis et al. 2007), identification of the NCAM^+^EpCAM^dim^ subset, possibly pinpoints an earlier MM-derived progenitor population (Fig. 9). Second markers that are expressed in all parts of the nephrogenic zone and are not detected on stromal cells potentially produce populations that include both MM-stem cells and a heterogeneous MM-derived progenitor population of the nephrogenic zone. This includes a wide variety of second marker combination, such as the rather small and discrete populations of NCAM^+^FZD7^+^ or NCAM^+^NTRK2^+^ cells we identified, but potentially also larger NCAM^+^CD24^+^ and NCAM^+^CD133^+^ cell populations (if indeed CD133 will be directly shown not to localize to stromal cells).

The rarities of putative MM-stem cells arising from condensates is demonstrated by triple FACS staining of these cell populations with EpCAM and analysis for those populations that totally lack epithelial differentiation (EpCAM^neg^). In all cases these were the smallest populations by comparison to EpCAM expressing fraction, showing NCAM^+^FZD7^+^EpCAM^−^, NCAM^+^NTRK2^+^EpCAM^−^ and NCAM^+^CD133^+^EpCAM^−^ cell fractions to be ≤1% of HFK cells, and NCAM^+^PSA-NCAM^+^EpCAM^−^ ∼2.5% of the cells. Interestingly, within the EpCAM^neg^ fraction there were NCAM^−^FZD7^+^ or NCAM^−^NTRK2^+^ but not NCAM^−^PSA-NCAM^+^ cells. These findings correlate with staining patterns in which FZD7 and NTRK2 also localize to loose mesenchyme (LM) while PSA-NCAM appears with condensation, possibly indicating the former fractions to arise from LM (Fig. 9).

In practice, cell sorting according to two positive markers and one negative is likely to be cumbersome and therefore eliminating EpCAM after positively selecting for a single marker that is expressed exclusively along the developmental stages of renal epithelia (MM, MM/UB-derived progenitors, developing and developed tubules but not stroma) might be more practical for sorting MM-enriched stem cells. In this setting, using an initial marker that localizes preferentially to the nephrogenic zone as opposed to a predominantly marker of differentiation is advantageous. One such potential combination includes the very small but consistent population of FZD7^+^EpCAM^−^ or PSA-NCAM^+^EpCAM^−^ cells. In any event, the relative paucity of stem/progenitor phenotypes highlights the need for early sorting of HFK cells according to marker molecules followed by their expansion in vitro rater than application of multipassage culture of unsorted heterogeneous HFK cells for cell selection (Loo et al. 2008).

The profiling of renal surface antigens initiated here forms the basis for exploring other markers and for investigating the function of suggested progenitor cell sub-populations in the renal context (Fig. 9). Clearly, this profiling of surface marker antigens needs to be extended so that a more complete picture of progenitor renal cell-cell interaction can be achieved. Moreover, new markers for renal stem/progenitor cells will be probably identified in the future and that the results obtained in this study will potentially need to be updated. Nevertheless, already at this stage, this study may prove particularly useful for potential application of cell-based therapies of renal diseases as well as for diagnosis of renal tumor stem cell populations, where the same markers may have similar relevance.

## References

[1] Bard JB, Gordon A, Sharp L, Sellers WI (2001). Early nephron formation in the developing mouse kidney.. J Anat.

[2] Boyle S, Misfeldt A, Chandler KJ, Deal KK, Southard-Smith EM (2008). Fate mapping using Cited1-CreERT2 mice demonstrates that the cap mesenchyme contains self-renewing progenitor cells and gives rise exclusively to nephronic epithelia.. Dev Biol.

[3] Brodbeck S, Englert C (2004). Genetic determination of nephrogenesis: the Pax/Eya/Six gene network.. Pediatr Nephrol.

[4] Challen GA, Martinez G, Davis MJ, Taylor DF, Crowe M (2004). Identifying the molecular phenotype of renal progenitor cells.. J Am Soc Nephrol.

[5] Cho EA, Dressler GR (2003). In The Kidney: From Normal Development to Congenital Disease..

[6] Cicero SA, Johnson D, Reyntjens S, Frase S, Connell S (2009). Cells previously identified as retinal stem cells are pigmented ciliary epithelial cells.. Proc Natl Acad Sci U S A.

[7] da Silva Meirelles L, Chagastelles PC, Nardi NB (2006). Mesenchymal stem cells reside in virtually all post-natal organs and tissues.. J Cell Sci.

[8] Dekel B, Reisner Y (2006). Applications of tissue engineering for the treatment of renal and uro-genital disease (chapter eds.)..

[9] Dekel B, Burakova T, Ben-Hur H, Marcus H, Oren R (1997). Engraftment of human kidney tissue in rat radiation chimera: II. Human fetal kidneys display reduced immunogenicity to adoptively transferred human peripheral blood mononuclear cells and exhibit rapid growth and development.. Transplantation.

[10] Dekel B, Shezen E, Even-Tov-Friedman S, Katchman H, Margalit R (2006a). Transplantation of human hematopoietic stem cells into ischemic and growing kidneys suggests a role in vasculogenesis but not tubulogenesis.. Stem Cells.

[11] Dekel B, Amariglio N, Kaminski N, Schwartz A, Goshen E (2002). Engraftment and differentiation of human metanephroi into functional mature nephrons after transplantation into mice is accompanied by a profile of gene expression similar to normal human kidney development.. J Am Soc Nephrol.

[12] Dekel B, Burakova T, Arditti FD, Reich-Zeliger S, Milstein O (2003). Human and porcine early kidney precursors as a new source for transplantation.. Nat Med.

[13] Dekel B, Metsuyanim S, Schmidt-Ott KM, Fridman E, Jacob-Hirsch J (2006b). Multiple imprinted and stemness genes provide a link between normal and tumor progenitor cells of the developing human kidney.. Cancer Res.

[14] Dominici M, Le Blanc K, Mueller I, Slaper-Cortenbach I, Marini F (2006). Minimal criteria for defining multipotent mesenchymal stromal cells. The International Society for Cellular Therapy position statement.. Cytotherapy.

[15] Durbeej M, Soderstrom S, Ebendal T, Birchmeier C, Ekblom P (1993). Differential expression of neurotrophin receptors during renal development.. Development.

[16] Esquela AF, Lee SJ (2003). Regulation of metanephric kidney development by growth/differentiation factor 11.. Dev Biol.

[17] Kim SS, Park HJ, Han J, Gwak SJ, Park MH (2007a). Improvement of kidney failure with fetal kidney precursor cell transplantation.. Transplantation.

[18] Kim SS, Gwak SJ, Han J, Park HJ, Park MH (2007b). Kidney tissue reconstruction by fetal kidney cell transplantation: effect of gestation stage of fetal kidney cells.. Stem Cells.

[19] Klein G, Langegger M, Goridis C, Ekblom P (1988). Neural cell adhesion molecules during embryonic induction and development of the kidney.. Development.

[20] Kobayashi A, Valerius MT, Mugford JW, Carroll TJ, Self M (2008). Six2 defines and regulates a multipotent self-renewing nephron progenitor population throughout mammalian kidney development.. Cell Stem Cell.

[21] Kreidberg JA, Sariola H, Loring JM, Maeda M, Pelletier J (1993). WT-1 is required for early kidney development.. Cell.

[22] Lazzeri E, Crescioli C, Ronconi E, Mazzinghi B, Sagrinati C (2007). Regenerative potential of embryonic renal multipotent progenitors in acute renal failure.. J Am Soc Nephrol.

[23] Loo D, Beltejar C, Hooley J, Xu X (2008). Primary and multipassage culture of human fetal kidney epithelial progenitor cells.. Methods Cell Biol.

[24] Metsuyanim S, Pode-Shakked N, Schmidt-Ott KM, Keshet G, Rechavi G (2008). Accumulation of malignant renal stem cells is associated with epigenetic changes in normal renal progenitor genes.. Stem Cells.

[25] Nishinakamura R (2003). Kidney development conserved over species: essential roles of Sall1.. Semin Cell Dev Biol.

[26] Oh SP, Li E (1997). The signaling pathway mediated by the type IIB activin receptor controls axial patterning and lateral asymmetry in the mouse.. Genes Dev.

[27] Pode-Shakked N, Metsuyanim S, Rom-Gross E, Mor Y, Fridman E (2008). Developmental tumorigenesis: NCAM as a putative marker for the malignant renal stem/progenitor cell population.. J Cell Mol Med.

[28] Rivera MN, Haber DA (2005). Wilms' tumour: connecting tumorigenesis and organ development in the kidney.. Nat Rev Cancer.

[29] Rosenblum ND (2008). Developmental biology of the human kidney.. Semin Fetal Neonatal Med.

[30] Roth J, Blaha I, Bitter-Suermann D, Heitz PU (1988). Blastemal cells of nephroblastomatosis complex share an onco-developmental antigen with embryonic kidney and Wilms' tumor. An immunohistochemical study on polysialic acid distribution.. Am J Pathol.

[31] Sagrinati C, Netti GS, Mazzinghi B, Lazzeri E, Liotta F (2006). Isolation and characterization of multipotent progenitor cells from the Bowman's capsule of adult human kidneys.. J Am Soc Nephrol.

[32] Salven P, Mustjoki S, Alitalo R, Alitalo K, Rafii S (2003). VEGFR-3 and CD133 identify a population of CD34+ lymphatic/vascular endothelial precursor cells.. Blood.

[33] Schmidt-Ott KM, Barasch J (2008). WNT/beta-catenin signaling in nephron progenitors and their epithelial progeny.. Kidney Int.

[34] Self M, Lagutin OV, Bowling B, Hendrix J, Cai Y (2006). Six2 is required for suppression of nephrogenesis and progenitor renewal in the developing kidney.. Embo J.

[35] Shmelkov SV, Butler JM, Hooper AT, Hormigo A, Kushner J (2008). CD133 expression is not restricted to stem cells, and both CD133+ and CD133- metastatic colon cancer cells initiate tumors.. J Clin Invest.

[36] Sugiyama T, Rodriguez RT, McLean GW, Kim SK (2007). Conserved markers of fetal pancreatic epithelium permit prospective isolation of islet progenitor cells by FACS.. Proc Natl Acad Sci U S A.

[37] Trzpis M, McLaughlin PM, Popa ER, Terpstra P, van Kooten TG (2008). EpCAM homologues exhibit epithelial-specific but different expression patterns in the kidney.. Transgenic Res.

[38] Trzpis M, Popa ER, McLaughlin PM, van Goor H, Timmer A (2007). Spatial and temporal expression patterns of the epithelial cell adhesion molecule (EpCAM/EGP-2) in developing and adult kidneys.. Nephron Exp Nephrol.

[39] Uchida N, Buck DW, He D, Reitsma MJ, Masek M (2000). Direct isolation of human central nervous system stem cells.. Proc Natl Acad Sci U S A.

[40] Weigmann A, Corbeil D, Hellwig A, Huttner WB (1997). Prominin, a novel microvilli-specific polytopic membrane protein of the apical surface of epithelial cells, is targeted to plasmalemmal protrusions of non-epithelial cells.. Proc Natl Acad Sci U S A.

[41] Weissman IL (2000). Translating stem and progenitor cell biology to the clinic: barriers and opportunities.. Science.

[42] Woolf AS (2001). The life of the human kidney before birth: its secrets unfold.. Pediatr Res.

[43] Xu ASL, Luntz TL, MacDonald JM, Kubota H, Hsu E (2000). Principles of Tissue Engineering, eds..

