# Correction: Expression of Stem Cell Markers in the Human Fetal Kidney

**DOI:** 10.1371/annotation/5c42ca40-0a01-4e4e-94e3-ccaa2cc54fc6

**Published:** 2011-03-17

**Authors:** Sally Metsuyanim, Orit Harari-Steinberg, Ella Buzhor, Dorit Omer, Naomi Pode-Shakked, Herzl Ben-Hur, Reuvit Halperin, David Schneider, Benjamin Dekel

The first author is missing a second affiliation. Sally Metsuyanim's second affiliation is: "The Mina & Everard Goodman Faculty of Life Sciences, Bar-Ilan University, Ramat-Gan, Israel."

